# The Impact of Centrifugation Devices and Collection Tubes on Fibrin Characteristics and Growth Factor Release Under High- and Low-Speed Protocols

**DOI:** 10.3390/dj13100476

**Published:** 2025-10-17

**Authors:** Oranit Bunyatratchata, Wutigri Nimlamool, Supatra Sangin

**Affiliations:** 1 Department of Restorative Dentistry and Periodontology, Faculty of Dentistry, Chiang Mai University, Chiang Mai 50200, Thailand; oranit.kate79@gmail.com; 2 Department of Pharmacology, Faculty of Medicine, Chiang Mai University, Chiang Mai 50200, Thailand; wutigri.nimlamool@cmu.ac.th

**Keywords:** platelet-rich fibrin, L-PRF, A-PRF+, centrifugation, blood collection tube

## Abstract

**Background:** Platelet-rich fibrin (PRF) is an autologous platelet concentrate (APC) produced through blood centrifugation. Despite the development of various centrifugation systems, protocol variability continues to pose challenges in selecting the optimal method. This study investigated the effects of three different centrifuges and collection tubes on the fibrin characteristics and growth factor release in leukocyte- and platelet-rich fibrin (L-PRF) and advanced platelet-rich fibrin plus (A-PRF+). **Methods**: Blood samples from six healthy female volunteers were processed using three centrifuges (Duo, IntraSpin, and LMC-3000) and three collection tubes (Pyrex, A-P, and silica-coated plastic) under high- (~700× *g* for 12 min) and low-speed (~200× *g* for 8 min) protocols. Fibrin clot weight and length were assessed. Growth factor release of platelet-derived growth factor-BB (PDGF-BB) and vascular endothelial growth factor (VEGF) was quantified using ELISA. Fibrin architecture was examined via scanning electron microscopy (SEM). **Results**: High-speed protocols tended to produce larger clots, whereas low-speed protocols generated smaller but more biologically active matrices. The IntraSpin and Duo centrifuges yielded greater clot dimensions and higher growth factor release than the LMC-3000. While tube type had no significant effect on growth factor levels, silica-coated tubes tended to produce the largest clots. The Pyrex tubes demonstrated comparable or superior growth factor release. **Conclusions**: PRF quality is influenced by centrifuge design, g-force, and tube material. Low-speed protocols with certified centrifuges are recommended, and FDA-approved glass tubes may provide a reliable alternative to reduce silica-related risks. Standardization and appropriate material selection are essential for consistent, safe, and effective regenerative outcomes.

## 1. Introduction

Autologous platelet concentrates (APCs) are biomaterials that have been utilized in several fields of medicine and dentistry for over three decades due to their growth factors, which can stimulate wound healing and promote soft and hard tissue regeneration [1]. Platelet-rich plasma (PRP), the first-generation APC, was introduced in 1998 to support bone reconstruction [2]. It contains high concentrations of platelets and growth factors and can be prepared in either liquid or gel form, making it beneficial in tissue healing. PRP has been successfully utilized in various clinical fields, including periodontal and bone regeneration, soft tissue augmentation, and temporomandibular joint disorders, as well as medical and aesthetic applications such as skin rejuvenation, hair restoration, orthopedic surgeries, and plastic surgeries [3,4,5]. Despite its broad clinical use, PRP presents notable limitations, as its preparation requires dual centrifugation and the addition of anticoagulants and activators, which prolong the protocol, may disrupt natural healing, and lead to variable clinical outcomes [6,7].

Therefore, a second-generation APC, known as leukocyte- and platelet-rich fibrin (L-PRF), was developed in 2001 [8]. It is prepared using a single centrifugation step in glass tubes without anticoagulants or activators, resulting in the formation of a three-dimensional fibrin clot that acts as a scaffold and reservoir for platelets, leukocytes, and growth factors. This fibrin enables the sustained release of key growth factors—including PDGFs, VEGF, bone morphogenetic protein (BMP), transforming growth factor-β (TGF-β), and epidermal growth factor (EGF)—which promote cell proliferation, differentiation, angiogenesis, and wound healing [1,9,10]. Its simple preparation and broad clinical integration further enhance its utility, particularly in oral surgery, endodontics, and implantology [11,12,13]. However, the solid or semi-solid nature of L-PRF limits its use in injectable applications, making different formulations suitable for specific purposes. PRP is preferred for liquid-based therapies, such as intra-articular or tendon injections, whereas PRF is advantageous as a clot or membrane, either alone or combined with biomaterials. As a membrane, PRF sustains growth factor release for up to 14 days, promotes angiogenesis, exhibits antibacterial activity, improves graft stability, and supports soft tissue healing [4,10].

The widespread adoption of this open-access technique, together with ongoing refinements in centrifugation protocols, has led to the development of several L-PRF derivatives. In 2014, advanced platelet-rich fibrin (A-PRF) was introduced based on the low-speed centrifugation concept (LSCC), which involves reducing centrifugal force and extending centrifugation time (~200× *g* for 14 min) using glass-based vacuum tubes [14]. This modification enhances growth factor concentration and promotes a more homogeneous distribution of platelets and leukocytes throughout the fibrin matrix [15]. A subsequent refinement, termed A-PRF+, further reduced centrifugation time (~200× *g* for 8 min), improving cell preservation, matrix porosity, and molecular integrity [16,17,18].

With the expanding clinical use of APCs, a wide range of commercially available centrifuges are now employed for PRF preparation. Previous studies have demonstrated that both centrifuge design and operational parameters—such as rotor angle, rotor radius, and vibration stability—significantly influence PRF characteristics. These factors affect key outcomes, including clot dimensions, cellular integrity, growth factor release kinetics, and fibrin polymerization [19,20,21]. Variations in centrifuge type, rotor configuration, and protocol settings can lead to marked structural and functional differences in PRF-based biomaterials.

Moreover, evidence indicates that the qualitative and quantitative properties of PRF are influenced not only by the centrifugation device but also by the type of collection tube used. Tube composition, surface coating, and manufacturing quality can affect coagulation dynamics, platelet distribution, and the retention or release profile of bioactive molecules. The significant impact of centrifugation tubes on PRF quality has also been emphasized in the previous study [21]. Early L-PRF studies used plain glass tubes to initiate clotting via the intrinsic coagulation cascade, whereas current practice employs various tube types—including glass, silica-coated plastic, and titanium—each exhibiting different levels of effectiveness. However, many of these tubes are approved only for laboratory diagnostic use, and their adoption in clinical PRF preparation largely stems from their availability and convenience in clinical and hospital settings [22].

Despite advancements in PRF protocols, the literature remains inconclusive regarding the optimal centrifugation parameters—such as rotor angle, rotor radius, and tube composition—with ongoing debate over their impact on PRF quality and reproducibility. Robust scientific evidence directly comparing different centrifugation devices and collection tubes across various protocols is still limited. This highlights the need for a clearer understanding of how centrifuge mechanics and tube material properties influence PRF formation to optimize its regenerative potential.

Therefore, this study aims to systematically investigate the effect of three commercially available centrifuges and three collection tube types on fibrin characteristics and growth factor release under high-speed (L-PRF) and low-speed (A-PRF+) protocols.

## 2. Materials and Methods

### 2.1. Study Design

In this study, blood samples were processed using three different centrifuges: the IntraSpin^TM^ PRF centrifuge (Intra-Lock, Boca Raton, FL, USA), the DUO Quattro^TM^ centrifuge (Process for PRF, Nice, France), and the LMC-3000 centrifuge (SIA Biosan, Riga, Latvia). Two protocols were applied to each device—the original L-PRF protocol (~700× *g* RCF max for 12 min) and the A-PRF+ protocol (~ 200× *g* RCF max for 8 min). The relative centrifugal force (RCF) values were calculated based on the rotor radius at maximum speed (RCF-max) to ensure accurate replication of manufacturer-recommended conditions for PRF preparation. The calculated RCF-max values for each centrifuge are presented in Table 1.

### 2.2. Subjects and Sample Collection

This study was approved on 31 July 2020 by the Human Experimentation Committee of the Office of Research Ethics, Faculty of Dentistry, Chiang Mai University (Approval No. 47/2020) and was conducted in accordance with the principles of the Declaration of Helsinki.

Participants were recruited voluntarily from the dental staff of the Faculty of Dentistry, Chiang Mai University. Prior to sample collection, all participants were informed of the purpose of the study, and written informed consent was obtained. The inclusion criterion was healthy female volunteers aged 20–40 years who were non-smokers. Exclusion criteria included: (i) systemic diseases such as diabetes mellitus or a recent history (within 6 months) of myocardial infarction (MI), cerebrovascular accident (CVA), immunocompromised conditions, chemotherapy, or radiotherapy; (ii) hematological disorders or bleeding abnormalities (e.g., platelet dysfunction or thrombocytopenia); (iii) use of anticoagulant or antiplatelet medication within the past 6 months; (iv) pregnancy or lactation; and (v) alcohol consumption. A total of six volunteers who met the inclusion criteria were enrolled, and the same individuals participated in both parts of the study.

### 2.3. Study Part 1: Centrifugation Analysis

#### 2.3.1. Preparation of Platelet Concentrates

Six healthy female volunteers were recruited; blood samples were obtained from the median cubital vein under aseptic conditions using a 24-gauge butterfly needle. Each participant underwent two separate blood collections, with 60 mL of blood drawn per session and transferred into 10 mL plain glass tubes (Process for PRF [A-P], Jiangxi Fenglin Medical Technology Co., Ltd., Nanchang, China). The collected blood samples (six tubes of 10 mL each) were immediately centrifuged to prepare L-PRF and A-PRF+ using three different centrifuges: IntraSpin, Duo, and LMC-3000. To ensure proper balance during centrifugation, 10 mL water-filled tubes were placed symmetrically opposite each sample tube in the rotor.

**Figure 1:**
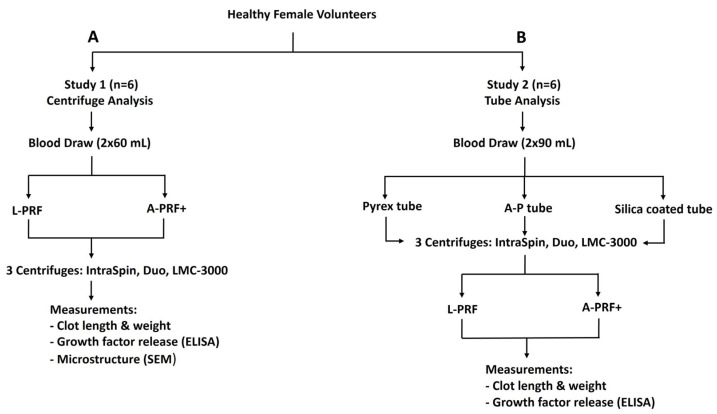
Flow diagram illustrating the study methodology: (**A**) Study 1—centrifuge analysis, and (**B**) Study 2—tube analysis.

Following centrifugation, PRF clots were gently retrieved using sterile tweezers and a smooth spatula to separate the red blood cell layer from the fibrin matrix, as previously described [21,23]. Each PRF clot was then placed on a sterile microscope slide, weighed, and measured for length using a Vernier caliper following established protocols [21,24]. Clot weight and clot length obtained from under each protocol were compared, and representative samples were subsequently processed for microscopic analysis. The experiment workflow for centrifuge analysis is illustrated in Figure 1A.

#### 2.3.2. Scanning Electron Microscopy (SEM)

Sample preparation for SEM was performed as previously described [23]. Briefly, PRF clots were gently compressed for two minutes on a sterile microscope slide to form membranes. The membranes were fixed in 2.5% glutaraldehyde, rinsed with phosphate buffer, and dehydrated through a graded ethanol series. Critical point drying and gold sputter coating were subsequently performed. The samples were examined using a scanning electron microscope (JSM-6610LV Series, JEOL Ltd., Tokyo, Japan), and images were acquired at 15–20 kV with magnifications ranging from ×2000 to ×5000 [19,21]. SEM images were taken under a single-blind protocol, with the examiner blinded to group allocation.

#### 2.3.3. Growth Factor Quantification

The release of two growth factors was quantified at specific time intervals (3 h, 6 h, 9 h, 1 day, 3 days, and 10 days) using enzyme-linked immunosorbent assay (ELISA). Fibrin clots were placed in 6-well plates containing 4 mL of Dulbecco’s Modified Eagle Medium (DMEM; Gibco, Thermo Fisher Scientific, Waltham, MA, USA) and incubated at 37 °C. At each designated time point, 4 mL of conditioned medium was collected and replaced with fresh DMEM. The collected samples were stored at −80 °C until further analysis [19]. ELISA kits (DuoSet, R&D Systems, Minneapolis, MN, USA) were used to quantify platelet-derived growth factor-BB (PDGF-BB; DY220, detection range: 15.60–1000 pg/mL) and vascular endothelial growth factor (VEGF; DY293B, detection range: 31.20–2000 pg/mL). Absorbance was measured at 450 nm with background correction at 570 nm using a microplate reader (Metertech, NanGang, Taipei, Taiwan). All samples from six biological replicates were analyzed in duplicate under each protocol.

### 2.4. Study Part 2: Centrifugation Tube Analysis

Six healthy female volunteers (the same cohort as in Part 1) participated in this experiment. Each underwent two venipunctures, with 90 mL of blood collected per session, yielding a total of 18 tubes (180 mL) per participant for analysis. Samples were immediately processed using three tube types: A-P tubes, silica-coated plastic tubes (BD Vacutainer^®^, Becton Dickinson, Franklin Lakes, NJ, USA), and Pyrex^®^ rimless glass test tubes (Corning, NY, USA), in combination with three centrifuges described in Part 1 to prepare L-PRF and A-PRF+. Following centrifugation, clot weight and length were measured according to the established protocol. For growth factor analysis, samples were examined using ELISA, with six biological replicates analyzed in duplicate under standardized conditions. The experimental design for tube analysis is illustrated in Figure 1B.

### 2.5. Statistical Analysis

Statistical analyses were performed using one-way ANOVA followed by post hoc multiple pairwise comparisons at a 95% confidence level. Data are presented as mean ± standard error of the mean (SEM), and a p-value < 0.05 was considered statistically significant. All statistical analyses were conducted using SPSS software, version 17 (IBM Corp., Armonk, NY, USA)

## 3. Results

### 3.1. Study Part 1: Centrifugation Analysis

#### 3.1.1. Macroscopic Analysis

**Figure 2:**
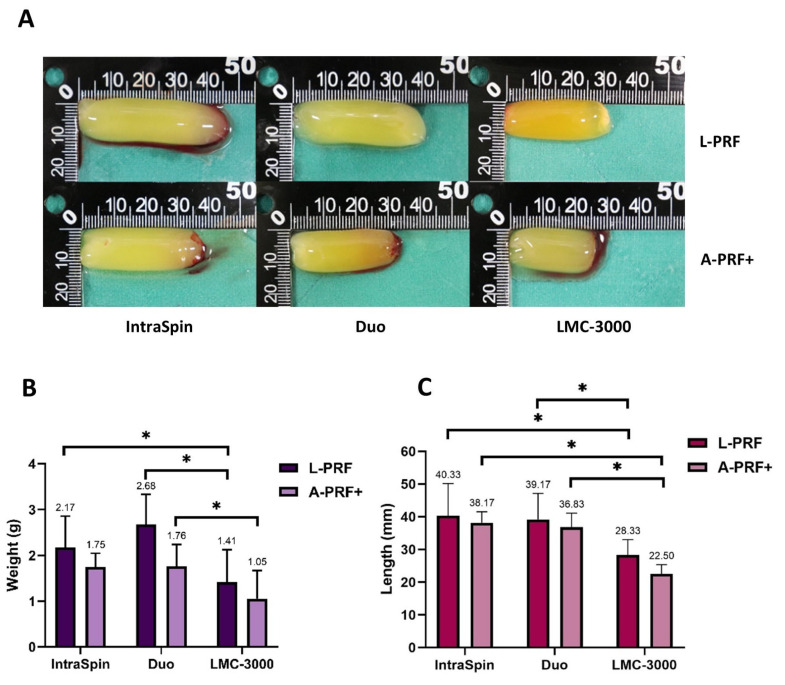
Macroscopic analysis of L-PRF and A-PRF+ prepared using three centrifuges (Intra-Spin, Duo, and LMC-3000). Representative images show (**A**) macroscopic clot morphology, (**B**) mean clot weight, and (**C**) mean clot length. Data are presented as mean ± SD, with statistically significant differences indicated by * (*p* < 0.05).

In the first experiment, the weight and length of fibrin clots were evaluated under high- and low-speed centrifugation protocols, with macroscopic outcomes presented in Figure 2. Overall, L-PRF clots produced at high-speed protocols were larger than those obtained under low-speed protocols (Figure 2A). Specifically, clot weight increased by 52% with the Duo, 34% with the LMC-3000, and 24% with the IntraSpin centrifuge compared with their respective low-speed counterparts. The Duo centrifuge produced significantly heavier clots than the LMC-3000 under both protocols, whereas no significant difference in clot weight was observed between the Duo and IntraSpin devices (Figure 2B). Regarding clot length, no significant differences were found between the IntraSpin and Duo centrifuges; however, both yielded significantly longer clots than those produced by the LMC-3000 (Figure 2C).

#### 3.1.2. SEM Analysis

**Figure 3:**
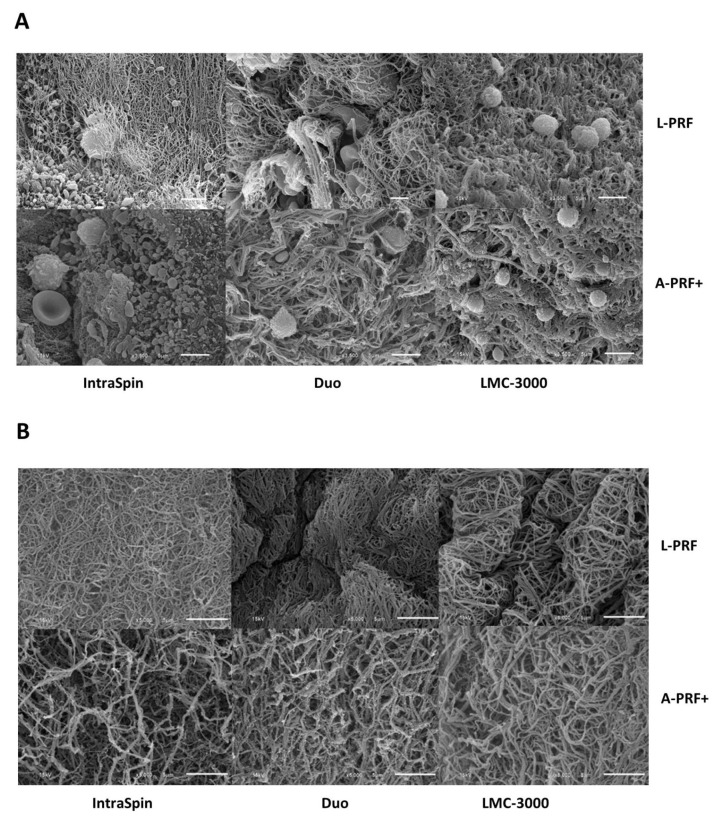
SEM analysis of L-PRF and A-PRF+ prepared using three centrifuges (Intra-Spin, Duo, and LMC-3000). Representative images show (**A**) fewer leukocytes, erythrocytes, and platelets in samples produced by the LMC-3000 compared with the IntraSpin and Duo centrifuges, and (**B**) a dense fibrin architecture in L-PRF versus a looser, more porous network with wider interfibrillar spaces in A-PRF+.

SEM analysis revealed distinct differences in cellular distribution and fibrin architecture among the centrifuges and protocols. The LMC-3000 centrifuge consistently produced fewer cellular components—including leukocytes, erythrocytes, and platelets— compared with the Intra-Spin and Duo centrifuges in both L-PRF and A-PRF+ preparations (Figure 3A). In terms of fibrin morphology, L-PRF produced by all three centrifuges exhibited a dense fibrin network with tightly packed fibers, whereas A-PRF+ displayed a looser and more porous structure with wider intercellular spaces. These findings highlight the influence of centrifugation protocols on fibrin network organization and clot porosity (Figure 3B).

#### 3.1.3. Release of Growth Factors

**Figure 4:**
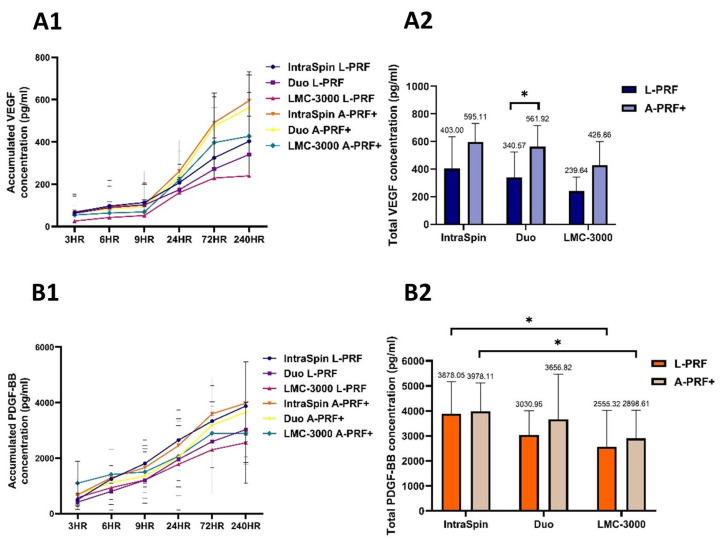
Growth factors release of L-PRF and A-PRF+ prepared using three centrifuges (Intra-Spin, Duo, and LMC-3000). Representative graphs show (**A1**) Accumulated VEGF and (**B1**) accumulated PDGF-BB released at multiple time points, and (**A2**) total VEGF and (**B2**) total PDGF-BB concentration. Data are presented as mean ± SD, with statistically significant differences indicated by * (*p* < 0.05).

The release profiles of VEGF and PDGF-BB were evaluated over a 10-day period, with measurements collected from 3 h to 10 days (Figure 4). Overall, A-PRF+ clots generated under low-speed centrifugation protocols exhibited higher concentrations of both growth factors compared with clots produced using high-speed settings. A statistically significant increase in VEGF release was observed only in clots prepared with the Duo centrifuge (Figure 4A2). In contrast, PDGF-BB release did not differ significantly between the IntraSpin and Duo centrifuges; however, IntraSpin clots, under both high- and low-speed protocols, demonstrated significantly higher PDGF-BB levels than those obtained with the LMC-3000 centrifuge (Figure 4B2). These findings suggest that both centrifugation protocol and device design influence growth factor release, with the low-speed concept promoting enhanced secretion—particularly for VEGF in the Duo centrifuge and PDGF-BB in the IntraSpin centrifuge.

### 3.2. Study Part 2: Centrifugation Tube Analysis

#### 3.2.1. Macroscopic Analysis

**Figure 5:**
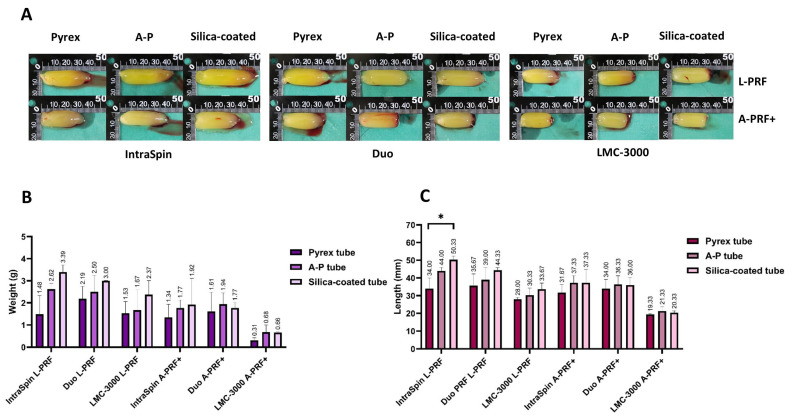
Macroscopic analysis of L-PRF and A-PRF+ prepared using three tubes (Pyrex, A-P, and silica-coated) in combination with three centrifuges (IntraSpin, Duo, and LMC-3000). Representative images show (**A**) macroscopic clot morphology of L-PRF and A-PRF+, (**B**) mean clot weight, and (**C**) mean clot length. Data are presented as mean ± SD, with statistically significant differences indicated by * (*p* < 0.05).

This study also evaluated the influence of different blood collection tubes on the macroscopic characteristics of platelet-rich fibrin matrices across three centrifuge systems, under both high- and low-speed protocols. Following centrifugation, clots produced under high-speed protocols were generally larger in both weight and length compared to those generated under low-speed conditions across all tube types (Figure 5A). Although no statistically significant differences in clot weight were observed among the tubes within the same centrifuge for either protocol, fibrin clots formed in silica-coated tubes tended to be the heaviest, followed by those from the A-P tubes and Pyrex tubes (Figure 5B). Regarding clot length, significant differences among tube types were found only with the IntraSpin centrifuge under the high-speed protocol, where the Pyrex tubes produced significantly shorter clots than silica-coated plastic tubes (Figure 5C). In contrast, no significant differences in clot length were observed among tube types when using the Duo or LMC-3000 centrifuges.

#### 3.2.2. Release of Growth Factors

**Figure 6:**
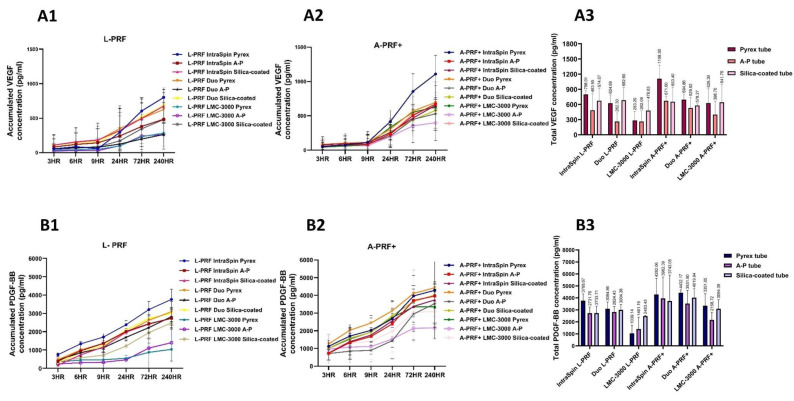
Concentrations of VEGF and PDGF-BB released from L-PRF and A-PRF+ prepared using three tubes (Pyrex, A-P, and silica-coated) in combination with three centrifuges (IntraSpin, Duo, and LMC-3000). Representative graphs show (**A1**) accumulated VEGF concentration and (**B1**) accumulated PDGF-BB concentration released from L-PRF at multiple time points. (**A2**) Accumulated VEGF concentration and (**B2**) accumulated PDGF-BB concentration released from A-PRF+ at multiple time points, and (**A3**,**B3**) total VEGF and PDGF-BB concentrations.

In terms of biological functionality, the release of growth factors PDGF-BB and VEGF was evaluated across different tube types in combination with three centrifuges (Figure 6). Although no statistically significant differences were observed among the tube types within each centrifuge system, it is noteworthy that the Pyrex tube—originally designed for scientific rather than clinical use—tended to show growth factor release levels comparable to those of the A-P tube, which is specifically manufactured for platelet centrifugation, as well as the silica-coated tube. Overall, both plain glass tubes performed similarly; however, the Pyrex tube exhibited slightly higher growth factor release compared with the A-P tube, although these differences were not statistically significant (Figure 6A3).

## 4. Discussion

Over the years, APCs have emerged as a promising regenerative tool due to their fibrin matrix and rich reservoir of growth factors. Recent studies indicate that the biological quality of PRF is strongly influenced by centrifugation parameters—particularly rotor orientation, g-force, and spin duration—which collectively determine fibrin architecture, cellular distribution, and growth factor release. In this present study, two fixed-angle centrifuges (IntraSpin and Duo) and one swing-out horizontal centrifuge (LMC-3000) were compared to evaluate their effects on fibrin characteristics and growth factor release under high-speed and low-speed protocols. Clot weight and length did not differ significantly between L-PRF and A-PRF+ when processed using the same centrifuge, consistent with previous findings [25]. However, L-PRF generally produced clots of greater weight and length compared with A-PRF+, suggesting that centrifugation speed and duration affect fibrin clot formation. This aligns with earlier studies reporting that higher centrifugation speed and longer duration contribute to the generation of larger fibrin clots [19,20,21].

Across all centrifuges evaluated, clot weight and length consistently increased under the high-speed protocol, with the Duo producing the largest clots, followed by the IntraSpin and LMC-3000. Both L-PRF and A-PRF+ clots generated with the Duo and IntraSpin centrifuges were significantly longer than those obtained with the LMC-3000, and the Duo consistently yielded heavier clots. These differences may be attributed to variations in centrifuge design, including vibration characteristics and rotor configuration. The LMC-3000, a swing-out laboratory centrifuge, exhibited greater vibration and reduced rotational stability, which may impair platelet entrapment and fibrin network organization. A previous study has demonstrated that increased vibration negatively affects clot architecture and cell distribution [19]. These findings indicate that even under identical speed and duration settings, centrifuge design can markedly influence clot dimensions and growth factor secretion profiles. Consistent with a previous report [25], no significant differences in clot size were observed between the Duo and IntraSpin centrifuges, both of which utilize fixed-angle rotors but differ in rotation angle and radius. Therefore, RCF should be carefully adjusted for each device to ensure equivalent forces on blood components, as discrepancies may otherwise lead to substantial differences in PRF structure. Overall, these results emphasize that despite protocol standardization, centrifuge design—including rotor angulation and radius— remains a critical determinant of clot size and quality [19,20,21].

Rotor type is another critical factor influencing PRF formation, as fixed-angle and swing-out horizontal designs can differentially affect fibrin clot development. The present findings align with a previous study reporting that L-PRF clots were significantly larger when produced using fixed-angle centrifuges [26]. However, other investigations have observed no significant differences in clot size between fixed-angle and horizontal rotor systems [27].

When growth factor release was compared, A-PRF+ matrices generally exhibited higher levels of PDGF-BB and VEGF than L-PRF. This difference was most pronounced with the Duo centrifuge, where VEGF release from A-PRF+ was significantly greater than that from L-PRF. These findings are consistent with previous studies [16,17], which also reported enhanced PDGF-BB and VEGF secretion from low-speed A-PRF+ matrices compared with high-speed L-PRF. The improved performance of A-PRF+ is attributed to low-speed centrifugation, which reduced fibrin density, facilitates cellular migration, and promotes a more gradual and sustained release of growth factors [2,14,18,28].

SEM analysis further supported these findings, revealing that lower-speed centrifugation protocols produced a looser and more porous fibrin network that facilitates cellular migration and promotes a more uniform distribution of platelets and leukocytes throughout the clot. This structure contributes to slower degradation and a prolonged release of growth factors, whereas higher-speed protocols generate denser fibrin networks that compact cells toward the bottom of the tube, leading to clustered cell distribution and faster degradation [14,17,23]. Consistent with these observations, the present study found that A-PRF+ clots exhibited a more porous fibrin architecture and a more sustained release of growth factors compared with L-PRF. Moreover, SEM analysis showed that platelets within both L-PRF and A-PRF+ clots remained predominantly in a normal, non-activated discoid state without extensive spreading or pseudopodia formation. Preservation of this morphology supports gradual growth factor release, with A-PRF+ demonstrating higher overall levels than L-PRF [15,18].

No significant differences in VEGF release were observed among the IntraSpin, Duo, and LMC-3000 centrifuges. This finding is consistent with previous reports indicating that VEGF levels remain statistically comparable over seven days between fixed-angle and swing-out horizontal centrifuges [26]. In contrast, PDGF-BB release from both L-PRF and A-PRF+ prepared with the IntraSpin was significantly higher than that obtained with the LMC-3000. This observation differs from earlier studies reporting that horizontal centrifugation produced a twofold increase in PDGF-BB and VEGF release from L-PRF membranes [29,30]. However, one of these studies also noted that fixed-angle centrifugation resulted in slightly greater FGF2 release compared with horizontal systems. These discrepancies suggest that centrifuge design, rotor orientation, and operational factors—including rotor radius, applied RCF, and vibration stability— collectively influence fibrin clot characteristics and may explain the variability in growth factor release reported across studies.

In the present study, the LMC-3000 centrifuge was compared with two commercially available fixed-angle centrifuges specifically designed for PRF preparation. The IntraSpin and Duo centrifuges are widely used and are considered reference devices in numerous experimental investigations [16,17,19,20,24,31,32,33,34,35,36]. Previous research has suggested that mechanical vibration may contribute to differences in PRF quality. The IntraSpin, an FDA-cleared device specifically designed for PRF preparation, exhibited the lowest vibration level, resulting in stable performance and high reproducibility of PRF clots while minimizing shear stress on platelets. The Duo, with a slightly larger rotor radius, demonstrated moderate vibration and produced generally stable clots, though minor variations in clot size were occasionally observed depending on tube balancing [19]. In contrast, the LMC-3000, a swing-out laboratory centrifuge—although CE marked for compliance with European safety standards—generates different acceleration vectors, and its larger rotor radius tends to increase vibration. These findings underscore that centrifuge vibration is an important yet often overlooked factor influencing the quality and consistency of PRF clots.

Another critical factor influencing PRF quality is the type of blood collection tube, which has been reported to affect final clot size as much as the centrifugation device itself [21]. Although no statistically significant differences in clot weight or growth factor levels were observed among Pyrex, A-P, and silica-coated tubes, a consistent trend indicated that silica-coated tubes produced the largest clots, likely due to the pro-coagulant properties of silica microparticles that accelerate clot initiation [35]. Significant differences in clot length among tube types were observed only when using the IntraSpin centrifuge, where Pyrex tubes produced significantly shorter fibrin clots than silica-coated tubes, while A-P tubes yielded intermediate results. In contrast, when using the Duo and LMC-3000 centrifuges, clot sizes remained relatively consistent across tube types, suggesting that device-specific centrifugal forces and rotor configurations may outweigh the subtle effects of tube composition.

Although tube type did not significantly affect growth factor release, Pyrex tubes tended to promote slightly higher levels when used with the IntraSpin and Duo centrifuges. Interestingly, despite producing significantly smaller clots—observed only with the IntraSpin system—Pyrex tubes, originally intended for laboratory diagnostics rather than clinical blood processing, demonstrated comparable or even higher PDGF-BB and VEGF release than silica-coated and A-P tubes. This finding suggests that clot size does not directly correlate with growth factor output, emphasizing that fibrin network quality and cellular entrapment play a more decisive role in regulating growth factor release kinetics, as similarly reported in previous studies [18,35].

Furthermore, PRF prepared in plain glass tubes has been shown to exhibit superior mechanical strength and biological safety compared with PRF obtained from silica-coated tubes [34]. Although not directly examined in this study, the influence of tube material on PRF biocompatibility warrants careful consideration. Previous reports have indicated that silica-coated tubes may release microparticles into the fibrin clot, potentially causing cytotoxic or inflammatory effects [37,38]. However, not all silica-coated tubes pose the same level of risk, as cytotoxicity varies depending on factors such as microparticle size, coating thickness, porosity, and surface adherence [34]. Until the biocompatibility of silica-coated tubes is fully established, the use of FDA-approved plain glass tubes, such as Pyrex^®^, is recommended as a safer and potentially more effective alternative for clinical PRF preparation. Collectively, these findings highlight that centrifuge design, operational speed, and tube composition substantially influence the structural integrity and biological performance of PRF matrices. They also underscore the need for greater standardization of PRF preparation protocols to optimize clinical outcomes, particularly regarding centrifugation parameters and tube selection. A limitation of this study is that only healthy female participants were included—a design choice intended to minimize biological variability and enhance reproducibility, but which limits generalizability. Additional limitations include the relatively small sample size and the evaluation of only two growth factors. Future studies should include larger and more diverse cohorts (e.g., both sexes, varying age groups, and individuals with systemic conditions), assess additional growth factors and cellular markers, and investigate the influence of centrifuge vibration on clot quality. Clinical trials are also warranted to validate the efficacy of PRF prepared using different centrifuges and tube types, thereby strengthening reproducibility and clinical applicability.

## 5. Conclusions

PRF quality is influenced by centrifuge design, g-force, and tube material. Low-speed protocols performed with certified centrifuges are recommended, while FDA-approved glass tubes may serve as a safer alternative to minimize silica-related risks. This study demonstrates that both centrifuge type and collection tube significantly affect PRF quality, including clot dimensions, fibrin architecture, and growth factor release. The IntraSpin and Duo centrifuges produced larger and more biologically active clots than the LMC-3000. Although tube type had a limited overall impact, both silica-coated and Pyrex tubes showed favorable trends in clot size and growth factor release. These findings emphasize the importance of standardizing centrifugation parameters and carefully selecting centrifuges and tube materials to ensure consistent, biologically optimal PRF for clinical applications.

## Figures and Tables

**Table 1:** Radius at maximum relative centrifugal force (RCF-max) and calculated high- and low-speed RCF values with corresponding revolutions per minute (RPM) for the three centrifuges used in this study, representing the ~700× *g* (L-PRF) and ~200× *g* (A-PRF+) protocol.

Centrifuge	IntraSpin	Duo	LMC-3000
	(Fixed Angle)	(Fixed Angle)	(Swing-Out Horizontal)
Radius at RCF-max	85 mm	110 mm	160 mm
High RCF (L-PRF) Low RCF (A-PRF+)	2700 RPM = 693× *g* 1450 RPM = 200× *g* (used in study 1500 RPM)	2385 RPM = 700× *g* (used in study 2400 RPM) 1300 RPM = 207× *g*	1978 RPM = 700× *g* (used in study 2000 RPM) 1057 RPM = 200× *g* (used in study 1100 RPM)

## Data Availability

Data is unavailable due to privacy.
